# Oxidative Damage Induced by Phototoxic Pheophorbide a 17-Diethylene Glycol Ester Encapsulated in PLGA Nanoparticles

**DOI:** 10.3390/antiox10121985

**Published:** 2021-12-13

**Authors:** Mariia R. Mollaeva, Elena Nikolskaya, Veronika Beganovskaya, Maria Sokol, Margarita Chirkina, Sergey Obydennyi, Dmitry Belykh, Olga Startseva, Murad D. Mollaev, Nikita Yabbarov

**Affiliations:** 1 N.M. Emanuel Institute of Biochemical Physics of Russian Academy of Sciences, 119991 Moscow, Russia; elenanikolskaja@gmail.com (E.N.); mariyabsokol@gmail.com (M.S.); fom.marg@mail.ru (M.C.); 2 JSC Russian Research Center for Molecular Diagnostics and Therapy, 117149 Moscow, Russia; Veronika.beganovckaya@bk.ru; 3 Department of Chemical and Pharmaceutical Technologies and Biomedical Products, Mendeleev University of Chemical Technology, 125047 Moscow, Russia; 4 Center for Theoretical Problems of Physicochemical Pharmacology, 119334 Moscow, Russia; obydennyj@physics.msu.ru; 5 Dmitry Rogachev National Medical Research Center of Pediatric Hematology, Oncology and Immunology, 117198 Moscow, Russia; md-mollaev@yandex.ru; 6 Institute of Chemistry of Komi Scientific Centre of the Ural Branch of Russian Academy of Sciences, 167982 Syktyvkar, Russia; belykh-dv@mail.ru; 7 Pitirim Sorokin Syktyvkar State University, 167001 Syktyvkar, Russia; om_startseva@mail.ru

**Keywords:** photodynamic therapy, photosensitizer, cellular uptake, oxidative stress, reactive oxygen species, cell death, nanoparticles, PLGA

## Abstract

Pheophorbide a 17-diethylene glycol ester (XL-8), is a promising high-active derivative of known photosensitizer chlorin e6 used in photodynamic therapy. However, high lipophilicity and poor tumor accumulation limit XL-8 therapeutic application. We developed a novel XL-8 loaded with poly(D,L-lactide-co-glycolide) nanoparticles using the single emulsion-solvent evaporation method. The nanoparticles possessed high XL-8 loading content (4.6%) and encapsulation efficiency (87.7%) and a small size (182 ± 19 nm), and negative surface charge (−22.2 ± 3.8 mV) contributed to a specific intracellular accumulation. Sustained biphasic XL-8 release from nanoparticles enhanced the photosensitizer photostability upon irradiation that could potentially reduce the quantity of the drug applied. Additionally, the encapsulation of XL-8 in the polymer matrix preserved phototoxic activity of the payload. The nanoparticles displayed enhanced cellular internalization. Flow cytometry and confocal laser-scanning microscopy studies revealed rapid XL-8 loaded nanoparticles distribution throughout the cell and initiation of DNA damage, glutathione depletion, and lipid peroxidation via reactive oxygen species formation. The novel nanoformulated XL-8 simultaneously revealed a significant phototoxicity accompanied with enhanced photostability, in contrast with traditional photosensitizers, and demonstrated a great potential for further in vivo studies.

## 1. Introduction

Photodynamic therapy (PDT) emerged around 120 years ago but still remains in high demand in modern medicine [1]. The current PDT application is significantly expanding; it is already applied in the treatment of cancer, atherosclerosis, microbial, and fungal diseases, the stimulation of immune response, and the reduction of adipogenesis and lipogenesis, etc. [2,3,4,5]. The PDT mechanism is based on the photosensitizer (PS) transition to the excited state under light irradiation and follows the formation of reactive oxygen species (ROS) and free radicals that result in apoptosis or necrosis [6]. In an excited triplet state, PS promotes the formation of singlet oxygen (type II mechanism) or superoxide anion, hydrogen peroxide, and hydroxyl radical (type I mechanism) [7]. Most researchers believe that type II mechanism is predominant in PDT [8]. However, recent data supports the idea that ROS-induced cell death mechanism may interfere with mitochondrial dysfunction, endoplasmic reticulum stress, and activation of pro-active “suicide” pathways [9,10,11]. Therefore, further research on the mechanisms underlying the phototoxic activity of PS may contribute to the identification of new targets and improve the effectiveness of PDT treatment.

PDT requires both light and PS; therefore, the design of novel light sources and PS is an interdependent process. The major categories of PDT light sources include filament bulbs, lasers, and light emitting diodes. The appropriate light source selection depends on the tumor location, PS, and delivered dose [12]. PS choice is a critical parameter for the successful PDT treatment. The most commonly used PSs are porphyrins, chlorins, bacteriochlorins, purpurins, metalloporphyrins, phthalocyanines, etc. [13,14]. Chlorin e6 (Ce6) is FDA approved second generation PS, which absorbs red light, characterized with relatively deep tissue penetration, in contrast with first generation PS [15,16]. Ce6 found application in esophagus, bladder, skin, head, and neck tumors treatment [17]. However, it as poor water solubility, precipitation in biological fluids, and increased dark toxicity limit Ce6 application [18]. Novel chlorin-based PSs often include peripheral hydrophilic moieties conjugated with macrocycle [17]. One of these derivatives is pheophorbide a 17-diethylene glycol ester (XL-8); a prospective amphiphilic PS [19]. However, it has relatively high lipophilicity limits bioavailability of XL-8. In the present study, we encapsulated XL-8 in nanoscale formulation to increase its bioavailability and accumulation selectivity and to reduce side effects via enhanced permeability and retention effect. Among a variety of different materials for modern nanodrugs design [20,21,22], we chose poly(D,L-lactide-co-glycolide) (PLGA), which is accepted for medical application and widely used as a material for surgical suture, orthopedics, and traumatology products and is also a carrier for drug delivery [23]. It is important that PLGA degrades in aqueous media in vitro and in vivo and forms lactic and glycolic acid monomers metabolized in the Krebs cycle [24,25]. 

In this study, we used Ce6 derivative XL-8; a promising PS agent to formulate XL-8-loaded PLGA nanoparticles (NPs) following characterization of their physicochemical properties, morphology, and in vitro drug release. The XL-8-NPs displayed high phototoxicity against HeLa, SK-OV-3, A549, 4T1, and MCF-7 cells. The XL-8-NPs caused main cellular damage via ROS generation, DNA breaks, and mitochondria failure. The present study provides a better understanding of mechanisms underlying the phototoxic activity of XL-8 and XL-8-NPs.

## 2. Materials and Methods

### 2.1. Materials 

Poly(D,L-lactide-co-glycolide) (PLGA polymer with carboxylic terminal group, 50/50 of inherent viscosity midpoint 0.2 dL/g; M_W_ 10,000–18,000) was purchased from LACTEL Absorbance Polymers (Birmingham, AL, USA). D-mannitol and polyvinyl alcohol (PVA, MW 30,000–70,000) were purchased from Sigma-Aldrich (St. Louis, MO, USA). Chloroform was purchased from Ruskhim (Moscow, Russia). Dimethyl sulphoxide (DMSO), Tween 80, and phosphate buffered saline (PBS) were purchased from Amreso (Solon, OH, USA). Trypsin and fetal bovine serum (FBS) were purchased from Hyclone (Logan, UT, USA). Ethylenediaminetetraacetic acid (EDTA), 2′-7′dichlorofluorescin diacetate (DCFH-DA), and paraformaldehyde were purchased from Serva (Heidelberg, Germany). DMEM and RPMI 1640 culture medium were purchased from Gibco (Waltham, MA, USA). 3-(4,5-dimethyl-thiazol-2yl)-2,5-diphenyltetrazoliumbromide (MTT), giemsa, mowiol, and o-phthaldialdehyde were purchased from Sigma-Aldrich (St. Louis, MO, USA). MitoSox Red was purchased from Invitrogen (Waltham, MA, USA). MitoStep was purchased from Immunostep (Salamanca, Spain). Annexin V-FITC/PI kit was purchased from Biolegend (San. Diego, CA, USA). TUNEL and Lipid Peroxidation (MDA) Colorimetric assay kits were purchased from Biovision (Milpitas, CA, USA). All other chemicals were used as HPLC grade or extra pure grade.

### 2.2. Experimental Methods

#### 2.2.1. Formulation of Nanoparticles

XL-8 was synthesized according to the transesterification method described previously [17]. XL-8-NPs were formulated via the single emulsion-solvent evaporation method with slight modifications. Briefly: 50 mg PLGA was dissolved in 5 mL chloroform containing 5 mg XL-8 and stirred during 10 min. After complete dissolution of substances, organic phase was added dropwisely to the 25 mL aqueous phase (1% *w/v* PVA) with constant stirring for 20 min. Then NPs dispersion was sonicated for 0.5 min at 20 kHz power and 50% amplitude (Labsonic U.B.Braun, Melsungen, Germany). Chloroform was evaporated for 40 min under reduced pressure (IKA HB10, Staufen im Breisgau, Germany). The particles were centrifuged at 14,000× *g* for 30 min, 4 °C and washed two times with deionized water to remove an unencapsulated XL-8 and surfactant (Beckman J2-21M, Beckman Coulter, Palo Alto, CA, USA). The formulated particles were resuspended in 10 mL water, filtered using a glass filter with a porosity of 111 μm, and 10% (*w/w*) D-mannitol was added to the suspension. Resulted NPs were freeze-dried (Alpha-I-5, Christ, Osterode am Harz, Germany) and stored at 4 °C.

#### 2.2.2. Size and Zeta Potential

Dynamic light scattering (DLS) and electrophoretic light scattering were applied to analyze the particles size, polydispersity index (PDI), and zeta potential using the Malvern Zetasizer system (Nano-ZS ZEN 3600, Malvern-Instruments, Worcestershire, UK). The responses were measured in triplicates.

#### 2.2.3. Loading Content and Entrapment Efficiency

The XL-8 loading content (LC) was determined by dissolving 4 mg lyophilized XL-8-NPs into 4 mL dimethylsulfoxide (DMSO) and subsequent absorption analysis at 668 nm. The XL-8 LC concentration was calculated according to the following equation (1):

(1) $$\mathrm{LC}=\frac{A_{\mathrm{DMSO}}\times \mathrm{Mw}\times V}{\varepsilon_{\mathrm{DMSO}}\times l\times m}\;\times \;100\%$$

where m is XL-8-NPs mass (g), V is DMSO volume (L), Mw is XL-8 molar mass equal to 662.75 g/mol, l is an optical path length (1 cm), ε_DMSO_ is the extinction coefficient of XL-8 in DMSO, equal to 83 500 L × mol^−1^ × cm^−1^, and A_DMSO_ is the absorbance of XL-8 in DMSO.

To calculate the entrapment efficiency (EE), 5 mg XL-8-NPs was dissolved in 1 mL distilled water with 0.1% (*m/v*) Tween 80 and centrifuged at 10,000× *g* for 5 min at room temperature. The supernatant was collected and lyophilized. Next, a sample was dissolved in 2 mL DMSO and analyzed using UV-Vis-spectrophotometry. EE was calculated according to the following Equation (2):

(2) $$\mathrm{EE}=\frac{m-m_{s}\;}{m}\;\times \;100\%$$

where m is the XL-8 amount encapsulated in 5 mg XL-8-NPs and m_s_ is the XL-8 amount in the supernatant.

#### 2.2.4. Nanoparticle Morphology

The morphology of XL-8-NPs was analyzed by transmission electron microscopy (TEM) (Osiris FEI, Hillsboro, OR, USA). The sample was prepared by placing one drop of XL-8-NPs (1 mg/mL in H_2_O) on a 3 mm copper grid covered with formvar film and dried for 30 min.

#### 2.2.5. In Vitro Release

In vitro release was analyzed using UV-Vis spectrophotometry. Briefly: 7 mg XL-8-NPs was resuspended in 1 mL PBS (0.01 M, pH 7.4) and transferred into a dark glass bottle containing 40 mL PBS (0.01 M, pH 7.4) and 0.1% (*m/v*) Tween 80. The sample was incubated in an orbital shaker (90× *g*) at 37 °C. At the defined time points, 1 mL aliquots was collected and replaced with 1 mL PBS. The collected samples were centrifuged at 3000× *g* for 15 min at 37 °C. The supernatant and sediment were removed and lyophilized. The resulting sediment and supernatant were dissolved in DMSO and analyzed, as described earlier.

#### 2.2.6. Cell Culture

The cancer cell lines: HeLa (human cervical carcinoma), SK-OV-3 (human ovarian adenocarcinoma), A549 (human lung adenocarcinoma), 4T1 (mice mammary gland adenocarcinoma), and MCF-7 (human breast adenocarcinoma) were maintained in Dulbecco’s-modified Eagle’s medium (DMEM), supplemented with 10% fetal bovine serum and gentamycin (50 μg/mL). Cells were grown in plastic 25 cm^2^ cell culture flasks at 37 °C in humidified atmosphere containing 5% CO_2_ (Sanyo, Osaka, Japan). Cells were seeded before reaching 80% confluence by detachment with trypsin/EDTA solution.

#### 2.2.7. XL-8 Photostability In Vitro

Fluorescence intensity of XL-8 was analyzed by flow cytometry to study XL-8 photostability in cells upon irradiation. HeLa cells at density 2 × 10^5^ in 6-well plates were treated with 120 nM XL-8 or XL-8-NPs for 2 h at 37 °C, followed by 20 min illumination with 25 mW/cm^2^ and a 660 nm light-emitting diode (LED). Cells were collected using trypsin/EDTA solution, washed twice with PBS, and analyzed. Fluorescence intensity was registered as the average value of fluorescence intensity (MFI) using flow cytometer Cyan ADP (Beckman Coulter, Brea, CA, USA), equipped with a solid-state laser (λex 635 nm) and 665/20 nm bandpass filter.

#### 2.2.8. Cytotoxic Activity

SK-OV-3, HeLa, A549, MCF-7, and 4T1 cells were seeded in 96-well plates (5000 cells per well) 24 h before the experiment and incubated under standard conditions. XL-8 or XL-8-NPs were added in triplets in the concentration range 0.016−1 μM (according to XL-8 concentration). To determine phototoxicity, cells were irradiated with 660 nm light-emitting diode for 20 min with a 25 mW/cm^2^ power LED and then incubated for 72 h. Cell survival was determined using standard MTT assay [26]: 50 μL MTT in DMEM (1 mg/mL) was added into each well. After cell incubation for 37 °C, the medium was removed and precipitated formazan crystals were dissolved in 100 μL DMSO. Following this, the absorption intensity of formazan was measured at 540 nm on a microplate reader. Cell viability was determined as percent of untreated control.

#### 2.2.9. Colony Formation Assay

HeLa cells were seeded at density 4 × 10^3^ (1 mL/well) in 6-well plates. A total of 24 h after seeding, cells were treated with 500 nM, 250 nM, and 125 nM XL-8 or XL-8-NPs for 2 h and irradiated with 25 mW/cm^2^ and 660 nm LED for 20 min 7 days of incubation. Afterwards, incubation colonies were gently washed twice with PBS and fixed for 5 min at room temperature with a mixture of 75% methanol/25% acetic acid and stained with 5% Giemsa for 30 min. The number of colonies was analyzed using ImageJ (National Institutes of Health, Bethesda, MD, USA).

#### 2.2.10. Intracellular ROS Registration Conditions

HeLa cells (1 × 10^5^) were seeded in 3 cm petri dishes and allowed to adhere overnight. Cells were treated with 100 nM XL-8 or XL-8-NPs for 2 h at 37 °C and irradiated with 25 mW/cm^2^ and 660 nm LED for 20 min. In order to select the conditions for ROS determination, we carried out three experiments: 20 µM 2,7-dichlorodihydrofluorescein diacetate (DCFH-DA) was added for 20 min immediately after PS treatment, after irradiation, and after harvesting cells. Following this, cells were rinsed twice with PBS, and intracellular ROS level was evaluated by flow cytometry (λex 488 nm, 530/40 nm bandpass filter).

#### 2.2.11. Intracellular ROS and Mitochondrial Superoxide Analysis

A qualitative ROS generation analysis was performed using flow cytometry. Briefly: 1 × 10^5^ HeLa cells were seeded in 3 cm petri dishes and allowed to adhere overnight. Following this, cells were treated with 100 nM XL-8 or XL-8-NPs for 2 h at 37 °C, stained with 20 µM DCFH-DA and 10 µM MitoSox Red in the dark separately for 20 min each, and irradiated with 25 mW/cm^2^ and 660 nm LED for 20 min. After the PBS wash, cells were collected and analyzed (λex 488 nm, 530/40 nm bandpass filter for DCFH-DA and 575/25 nm bandpass filter for MitoSox Red).

#### 2.2.12. ROS and XL-8-NPs Subcellular Localization

The intracellular XL-8-NPs localization in HeLa cells was analyzed by confocal laser scanning microscopy (CLSM). A total of 1.9 × 10^4^ (1 mL/well) HeLa cells were seeded on the coverslips in 24-well plates for 24 h. The next day, cells were incubated for 2 h in serum free media and treated with 120 nM XL-8-NPs for another 2 h, rinsed three times with PBS, and stained with 2.2 µM hoechst 33,342. Following this, cells were fixed in 2% paraformaldehyde, rinsed with PBS, and embedded in mowiol. XL-8-NPs and hoechst 33,342 fluorescence intensity was observed by using a Carl Zeiss Cell Observer Z1 confocal microscope with 100× magnification (Jena, Germany). Pictures were finally processed for selection of various color combinations using Photoshop software (Adobe, Mountain View, CA, USA).

HeLa cells were plated and treated, as mentioned above, for live ROS and mitochondrial superoxide visualization. After XL-8 or XL-8-NPs treatment, cells were stained with 20 µM DCFH-DA and 10 µM MitoSox Red for 20 min in the dark and irradiated with a 660 nm LED with 25 mW/cm^2^ power for 20 min. The slides were rinsed, embedded in mowiol, and analyzed immediately by CLSM.

#### 2.2.13. Mitochondrial Membrane Potential Assay

Changes in mitochondrial membrane potential (MMP) were analyzed by staining cells with DilC1(5) fluorescent probe. 2 × 10^5^ HeLa cells were seeded in 6-well plates. After XL-8 or XL-8-NPs treatment and light irradiation, cells were rinsed with PBS, harvested, and incubated with 10 μM DilC1(5) for 15 min at 37 °C. After incubation, fluorescence intensity was analyzed by flow cytometry (λex 635 nm, 665/20 nm bandpass filter).

#### 2.2.14. Apoptosis Assay

Necrotic and apoptotic populations were analyzed using annexin V-FITC/propidium iodide (PI) staining. Briefly, 1 × 10^5^ HeLa cells were seeded in petri dishes and incubated overnight. After PDT treatment (40 min incubation with the PSs followed irradiation), cells were rinsed, harvested, and incubated with 10 μL (5 μg/mL) annexin V-FITC and 10 μL PI (20 μg/mL). After 15 min of incubation, cells were rinsed and immediately analyzed by flow cytometer (λex 488 nm, 613/20 nm bandpass filter for PI and 530/40 for annexin V-FITC).

#### 2.2.15. TUNEL Assay

HeLa cells were seeded on the 96-well plates, 4 × 10^3^ per well. The next day, cells were incubated with different concentrations of XL-8 or XL-8-NPs (120 nM, 60 nM, 30 nM) for 2 h and irradiated. The evaluation of DNA breaks in cells was performed by flow cytometry using a TUNEL kit (λex 488 nm, 575/25 bandpass filter nm).

#### 2.2.16. Lipid Peroxidation (MDA) Assay

Lipid peroxidation level was assessed using MDA assay kit. Briefly, HeLa cells were seeded in 96-well plates and incubated overnight. The next day, cells were treated with XL-8 or XL-8-NPs, irradiated, as described earlier, and incubated for 24 h. Treated cells were harvested, homogenized on ice in 300 µL MDA lysis buffer, and centrifuged for 10 min at 10,000× *g*. The supernatant was collected and incubated with 600 µL TBA reagent for 60 min at 95 °C. The samples were cooled on ice for 10 min, and absorption spectra of the MDA-TBA adduct was recorded at 532 nm.

#### 2.2.17. Reduced Glutathione (GSH) Assay

The analysis of the reduced glutathione level was performed according to the method by Hedley et al. [27]. Briefly, 5×10^5^ per well HeLa cells were seeded in 96-well plates and incubated overnight. XL-8 or XL-8-NPs were added to adhered cells at different concentrations (120 nM, 60 nM, and 30 nM), incubated for 2 h, and irradiated, as described earlier. After 24 h, incubation cells were harvested and stained with 0.5 mM o-phtaldialdehyde for 5 min at room temperature. The fluorescence intensity was determined by flow cytometry (λex 488 nm, 530/40 bandpass filter).

#### 2.2.18. In Vivo Antitumor Activity

The experimental procedures in mice were approved in the N.M. Emanuel Institute of Biochemical Physics Ethics Committee for use of experimental animals and performed according to the guidelines of European Medicines Agency, Amsterdam, Netherlands. Female BALB/c Nude mice (body weight 20–22 g) were supplied by the Pushchino animal facility (Russia). The animals were kept under controlled aseptic conditions (temperature of 22 ± 2 °C and relative humidity of 50 ± 10%), with free access to sterilized water and standard pellet feed.

Antitumor activity of XL-8-NPs and XL-8 was evaluated using HeLa tumor-bearing mice. The anticancer treatments were performed when the tumors reached approximately 100 mm^3^. The mice were randomly divided into three groups (*n* = 3): XL-8 (10 mg/kg), XL-8-NPs (200 mg/kg), and saline. The mice were injected once with samples via lateral tail vein. After 2 h of treatment, mice were irradiated with 660 nm LED with 0.3 W/cm^2^ for 5 min. Tumor sizes and mouse body weights were recorded every three days, and tumor volumes were calculated according to the following formula:

(3) $$Volume=\frac{lenght\times width^{2}}{2}$$

Tumor-bearing mice were sacrificed on day 25 after initial drug treatment.

#### 2.2.19. Statistical Analysis

The data visualization and statistical analysis were performed in OriginPro (OriginLab Corporation, Northampton, MA, USA) and Excel (Microsoft Corporation, Redmond, WA, USA). The results are represented as mean ± standard deviation. One-way analysis of variance was applied to determine the significant difference. *p* < 0.05 was considered as a significant.

## 3. Results

### 3.1. Nanoparticles Formulation

**Figure 1:**
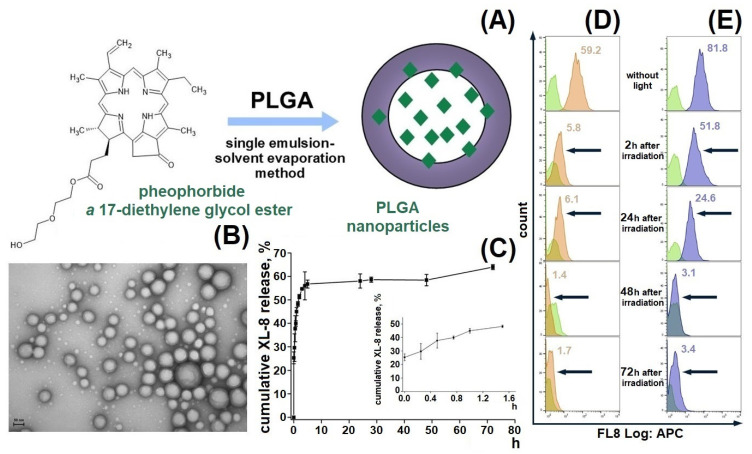
Formulation scheme of XL-8-loaded PLGA NPs (**A**) and TEM micrograph of XL-8-loaded NPs. Scale bar corresponds to 50 nm (**B**). In vitro release of XL-8-loaded NPs in PBS (pH 7.4). Each point shows mean ± S.D. (*n* = 3) (**C**). Flow cytometry analysis of HeLa cells treated with XL-8 (120 nM; orange) (**D**) and XL-8-NPs (120 nM; blue) (**E**) (not treated cells; green), irradiated and analyzed after 2 h, 24 h, 48 h, and 72 h. MFI of cells treated with XL-8 (orange numbers) and XL-8-NPs (blue numbers).

We designed a passive delivery system based on XL-8 loaded PLGA NPs to increase XL-8 bioavailability and internalization specificity (Figure 1A). The formulated NPs displayed a spherical morphology, with a relatively small size of 182 ± 19 nm, a low polydispersity index of 0.129, negative zeta potential of −22.2 ± 3.8 mV (Figure S1), and high LC (4.6%) and EE (87.7%) of XL-8. Low aggregation prone, small size, and negative charge are favorable parameters for specific cell endocytosis, and high PS content is the main property indicated in the NP perspectives in PDT [21,28].

### 3.2. Morphology Studies

A TEM micrograph demonstrated the spherical shape of NPs with narrow size distribution (Figure 1B), lack of aggregates, and correlation with the dynamic light scattering data (Figure S1).

### 3.3. In Vitro XL-8 Release

The XL-8 release profile demonstrated a biphasic pattern: initial burst release during the first 5 h, followed by a prolonged sustained phase up to 73% in 70 h (Figure 1C), which agreed with previous results [29,30,31] and was often observed in NPs smaller than 200 nm compared to microparticles [32,33]. The initial burst release occurs due to XL-8 detachment from NPs surface or release of XL-8 molecules placed near the inner surface, easily accessible by hydration [34]. The second release phase showed slow XL-8 diffusion from PLGA pores or cracks formed due to hydration and degradation process.

### 3.4. XL-8 Photostability In Vitro

We tested XL-8-NPs photostability in comparison to XL-8 by evaluating its fluorescence intensity in HeLa cells after LED irradiation for 20 min. According to the literature data, we used 120 nM XL-8, which does not affect cell viability [35]. Our results demonstrated a time-dependent decrease in XL-8 and XL-8-NPs fluorescence intensity, as shown in Figure 1D,E. However, we observed a higher fluorescence intensity of XL-8-NPs in comparison to XL-8 at 2 h and 24 h, which may be explained by XL-8 prolonged release from NPs. According to these results, we concluded that XL-8 encapsulation in NPs preserves XL-8 photostability for 24 h and its safety.

### 3.5. Intracellular Internalization of XL-8-NPs

**Figure 2:**
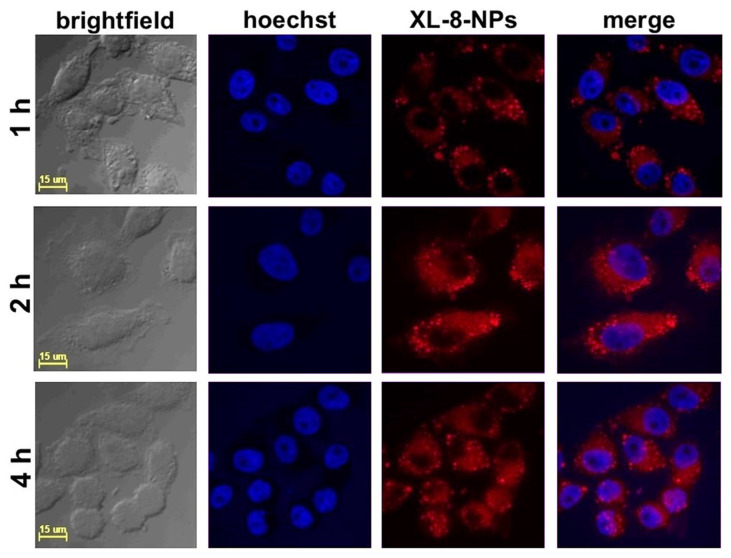
Confocal fluorescence images of HeLa cells treated with XL-8-NPs (120 nM) for 1, 2, and 4 h, with hoechst 33,342.

According to the literature data, the effective cellular uptake of NPs occurred within 4 h and was further replaced by saturation of internalization [36]. We studied the intracellular accumulation efficacy of NPs (120 nM according XL-8) after 1, 2, and 4 h of incubation. The NPs demonstrated highly efficient accumulation in HeLa cells after 2 h of incubation (Figure 2).

The quantitative fluorescence analysis (Figure S2) of confocal images performed with a protocol from QBI (The University of Queensland, St. Lucia, Australia) [37] revealed the lowest XL-8 fluorescence intensity after 1 h of incubation. The fluorescence values after 2 h and 4 h of staining characterized with insignificant difference. These results agreed with previously reported data concerning NPs accumulation in cancer cells [38,39]. Summarizing visual and quantitative fluorescence analysis results, we applied 2 h of XL-8 and XL-8-NPs incubation in further experiments.

### 3.6. Cytotoxicity of XL-8 and XL-8-NPs In Vitro

An ideal PS has to be a highly toxic upon irradiation and safe in the dark. We assessed the light and dark cytotoxicity of XL-8 and XL-8-NPs using standard MTT-test against HeLa, SK-OV-3, A549, MCF7, and 4T1 cells [40,41].

**Figure 3:**
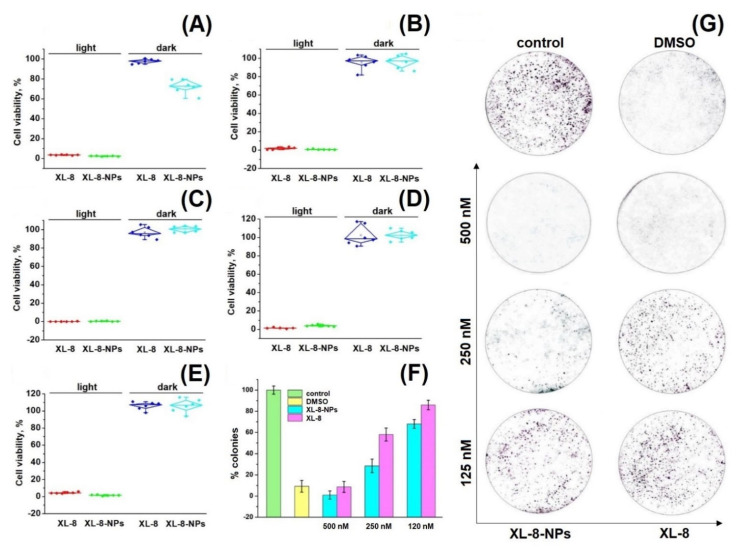
Photo- and cytotoxicity of XL-8 (500 nM) and XL-8-NPs (500 nM) analyzed by MTT assay. The percentage of cell viability was determined relative to viable control cells. SK-OV-3 cells (**A**), A549 cells (**B**), HeLa cells (**C**), MCF7 cells (**D**), and 4T1 cells (**E**) measured at 72 h after treatment. The quantification of colony formation rate in HeLa cells after treatment with 10% DMSO and XL-8- or XL-8-NPs-based PDT (**F**). Giemsa-stained plates of colony formation assay carried out on HeLa cells (**G**).

Preliminary cytotoxicity studies of XL-8 allowed to adjust optimal PS concentrations and irradiation regimen (data not shown). As shown in Figure 3A–E, XL-8 and XL-8-NPs remained up to 80% of cell viability in the absence of light, indicating low dark toxicity. While using irradiation, XL-8 and XL-8-NPs exhibited a strong cytotoxic effect against all examined cells, suggesting that XL-8-NPs may become a promising photosensitizer for the treatment of a wide range of tumor diseases (Table S1). Since HeLa cells demonstrated that they were the most sensitive to XL-8 and XL-8-NP phenotypes, we used this cell line in further experiments.

Colony formation analysis shows the ability of a single cell to survive under PDT exposure and form a colony [42]. Our data demonstrated the efficiency of both XL-8- and XL-8-NPs to inhibit colony formation in a dose-dependent manner. Nevertheless, the growth rate inhibition ability of XL-8-NPs was considerably higher compared to free XL-8 (Figure 3F,G). It is noteworthy that XL-8 and XL-8-NPs, at high concentrations, evoked a significant reduction in HeLa colony formation, while the control group formed large colonies. Summarizing, XL-8 entrapment into the PLGA matrix showed an enhanced light-toxicity against HeLa and MCF-7 cells.

### 3.7. ROS Formation Analysis

Since the PDT mechanism was based on the oxidative stress, we analyzed the ability of XL-8 to induce ROS generation. We applied DCFH-DA dye, which was cleaved by intracellular esterase into non-fluorescent DCFH, followed by conversion to green fluorescent DCF in the presence of intracellular ROS [43], to determine ROS level after XL-8 (100 nM) and XL-8-NPs (100 nM) treatment and irradiation.

**Figure 4:**
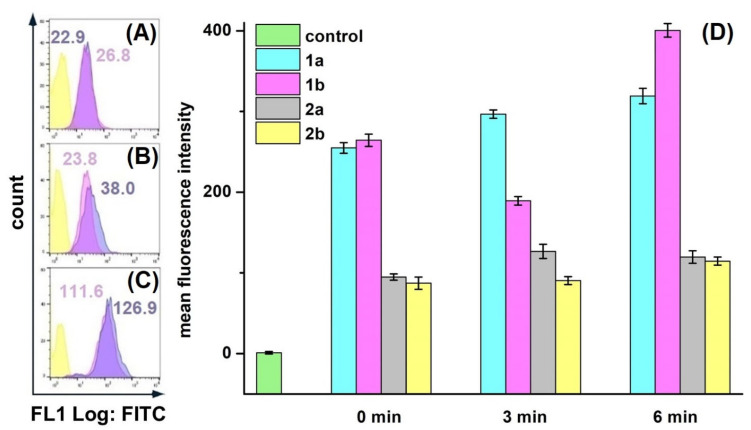
Different conditions of ROS registration in HeLa cells treated with XL-8 (100 nM; blue) and XL-8-NPs (100 nM; violet) (not treated cells; yellow) using DCFH-DA. Fluorescent dye added: after harvesting cells (**A**), irradiation (**B**), and PS treatment (**C**). MFI of cells treated with XL-8 (blue numbers) and XL-8-NPs (violet numbers). Evaluation of ROS (1) and superoxide anion (2) levels in HeLa cells, treated with XL-8 (a) and XL-8-NPs (b) (**D**).

We decided to optimize conditions of intracellular ROS detection, since protocols available in the literature varied greatly. Cells were stained with DCFH-DA after cell harvesting (Figure 4A), after irradiation; (Figure 4B), and before irradiation (Figure 4C). In XL-8 and XL-8-NPs treated samples, cells stained before irradiation and after harvesting displayed the similar fluorescence levels of DCF, comparable with unstained cells. It is likely that the low MFI levels may be associated with the time-dependent process of DCFH-DA ester bond cleavage and poor DCF fluorescence in the ROS presence. Another important stage is cells harvesting by detachment with trypsin, followed by staining with DCFH-DA, which is a long-term process, possibly increasing additional cellular stress and hampering accurate intracellular ROS registration. In contrast, cells stained with DCFH-DA before irradiation facilitated the efficient ROS registration. Therefore, we applied the described staining methodology in further experiments to improve ROS registration accuracy.

**Figure 5:**
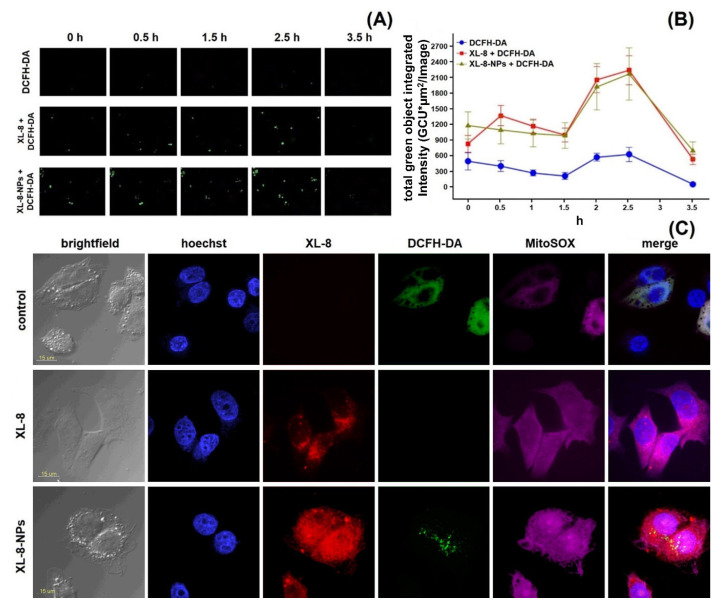
ROS formation kinetics in HeLa cells. Control cells were stained with DCFH-DA and irradiated. Two identical plates were treated and irradiated with XL-8 (100 nM) and XL-8-NPs (100 nM). Images were acquired every 30 min. Captured images from IncuCyte using a ×20 objective; 200 μm scale bars (**A**). At the end of analysis, an automatic real-time assessment was performed using IncuCyte, measured as green object integrated intensity for all cells stained green with DCFH-DA, allowing generating of graphical data (**B**). Fluorescent images of HeLa cells. Subcellular localization was investigated after treatment with XL-8 (100 nM) and XL-8-NPs (120 nM), hoechst 33,342, DCFH-DA, and MitoSox Red (**C**).

HeLa cells displayed an intensive DCF green fluorescence after treatment with both formulations, followed by irradiation, evidencing the intracellular ROS formation. XL-8 demonstrated the strongest fluorescence intensity at 3 and 5 min after irradiation, indicating ROS level elevation. The XL-8-NPs induced the higher signal after 6 min, evidencing the sustained XL-8 release and predominant particles accumulation in tumor cells (Figure 4D). DCF fluorescence live analysis (Figure 5A,B) confirmed the most intensive ROS generation within the first 2.5 h after XL-8 and XL-8-NPs treatment and irradiation. Our results agreed with the previous report by Gao et al., who described PDT-induced ROS as peaking at 3 h after irradiation [7].

Considering the DCFH-DA low fluorescence specificity to particular ROS, we analyzed the XL-8-based vehicles’ ability to induce mitochondrial superoxide formation using a MitoSox Red fluorescent probe (Figure 4D). The fluorescence intensity of MitoSox Red displayed the similar profiles of superoxide anion and ROS formation. However, XL-8 induced stronger superoxide anion generation than XL-8-NPs (Figure 5C). This effect may be explained by the lipophilic nature of XL-8, which promoted fast mitochondrial accumulation [44].

We concluded that both XL-8 and XL-8-NPs effectively induced ROS generation, but XL-8 encapsulation into NPs elevated total intracellular internalization and stabilized XL-8 by a prolonged release and ROS generation.

### 3.8. Subcellular Localization Study

According to the time-resolved live fluorescence analysis, XL-8 and XL-8-NPs displayed a similar ability to induce oxidative stress during the initial phase after irradiation (Figure 5A,B). The comparative ability of XL-8-NPs to generate ROS may be explained by a slow XL-8 release from NPs.

Furthermore, we performed intracellular visualization of live HeLa cells after 2 h incubation with XL-8 or XL-8-NPs, immediately after irradiation by CLSM. The NPs (red signal) mainly displayed perinuclear localization (Figure 5C); the predominant dot-shaped fluorescence pattern indicated the endo-lysosomal accumulation, evidencing the endocytic nature of internalization, while sparse homogenous areas corresponded with the gradual XL-8 release from polymer matrix. We also revealed a partial overlapping of MitoSox Red, DCF, and XL-8 fluorescence, suggesting the PS accumulation in mitochondria, which agreed with previous reports that described bacteriochlorins and phtalocyanins intracellular localization [45,46]. Relatively weak intranuclear MitoSox Red fluorescence may be explained by a time-dependent redistribution of dye [47,48]. XL-8-NPs lacked co-localization with nuclei stained with hoechst 33,342 but partially merged with DCFH-DA in the perinuclear area. XL-8 demonstrated weak intracellular entrapment but was able to stimulate a prominent MitoSox Red fluorescence. Interestingly, samples treated with XL-8 and XL-8-NPs almost lacked DCFH-DA fluorescence compared to control cells.

These results demonstrated a high XL-8-NPs ability to internalize, redistribute along organelles, and, despite sustained release, stimulate oxidative stress at the same level as free XL-8 during the initial PDT phase.

### 3.9. Apoptosis Study

**Figure 6:**
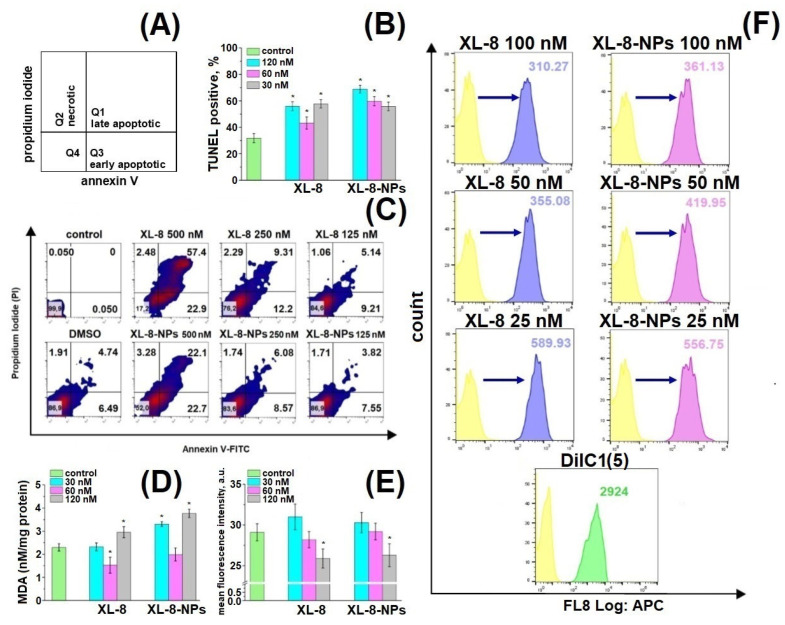
Schematic distribution of late apoptotic, early apoptotic, and necrotic populations in the corresponding quadrants (**A**). Percentage of TUNEL-positive HeLa cells treated with XL-8 and XL-8-NPs and irradiated. * *p* < 0.05 compared with the TUNEL treated group (control) (**B**). Apoptotic and necrotic HeLa cell populations after XL-8- or XL-8-NPs-based PDT. DMSO corresponds to 2% solution in DMEM (**C**). MDA analysis in HeLa cells. * *p* < 0.05 compared with the control group (TBA treated) (**D**). MFI of HeLa cells treated with XL-8 and XL-8-NPs, irradiated, and stained with o-phthaldialdehyde. * *p* < 0.05 compared with the control group (o-phthaldialdehyde treated cells) (**E**). Mitochondrial membrane potential (MMP, ΔΨm) of HeLa cells treated with XL-8 and XL-8-NPs, irradiated (**F**). The results are shown as the mean ± S.D. (*n* = 3).

We applied annexin V-FITC/PI double staining to analyze cell death induced by XL-8 and XL-8-NPs (Figure 6A) [49,50].

We observed a dose-dependent character of PDT-induced damage and formation of late and early apoptic populations (Figure 6C). Up to 81.4% of cells were at different apoptosis phases after XL-8 treatment at a concentration of 500 nM compared to XL-8-NPs (43.7%); the late to early apoptotic populations ratio after XL-8 treatment was significantly higher than XL-8-NPs, but dose reductions of up to 250 nM and 125 nM mitigated this effect. The pronounced toxicity difference at high doses could be explained by specificities of intracellular accumulation kinetics and interaction with membrane compartments of lipophilic XL-8 and hydrophilic XL-8-NPs.

### 3.10. TUNEL Assay

TUNEL assay revealed a dose-dependent manner of DNA fragmentation induced by ROS formation after XL-8- and XL-8-NPs-based PDT (Figure 6B) [51,52]. XL-8-NPs (120 nM) treatment generated an increased number of TUNEL positive cells (75.99%) compared to XL-8 (55.89%) at the same concentration. We assumed that uptake of XL-8-NPs via endocytosis and ROS formation in the perinuclear area may contribute to excessive DNA damage. Thus, a higher percentage of TUNEL positive cells evidenced that NPs-based delivery provided better conditions for intracellular ROS generation and effective PDT-induced DNA damage compared to free XL-8.

### 3.11. Mitochondrial Membrane Potential

Mitochondria play a major role as cellular power plants and regulate cell survival and death. Changes in mitochondrial membrane potential (MMP) may evidence the organelle failure and lead to apoptosis [53]. We applied a permeable lipophilic fluorescent cationic dye DiIC1(5) as a marker of mitochondrial membrane depolarization (∆Ψm) [54]. Viable mitochondria with high membrane potential actively accumulated DiIC1(5), demonstrating a strong fluorescence, while in cells treated with XL-8 and XL-8-NPs mitochondria displayed dose-dependent fluorescence intensity decrease (Figure 6F). The mitochondrial depolarization was a bit stronger in samples treated with 100 nM, 50 nM XL-8, and 25 nM XL-8-NPs, evidencing the oxidative stress and mitochondrial dysfunction. Overall, these results agreed with previous reports and described Ce6 accumulation in mitochondria [55,56], which may indicate the apoptosis induction via mitochondrial depolarization.

### 3.12. GSH and MDA Assay

Furthermore, we analyzed the cytoplasmic oxidative stress markers to assess consequences of XL-8- and XL-8-NPs-based PDT. GSH supports intracellular RedOx homeostasis [57]. PDT-induced GSH depletion may imbalance this system and trigger apoptosis [58]. O-phthaldialdehyde interacts with intracellular GSH, forming fluorescent derivative. Both XL-8 and XL-8-NPs decreased GSH in a dose-dependent manner and displayed similar activity (Figure 6E).

Along with GSH, we analyzed the level of malondialdehyde (MDA), one of the most prevalent and mutagenic lipid peroxidation products [59]. We determined the MDA concentration in HeLa cells after XL-8- and XL-8-NPs-based PDT. Both formulations induced lipid peroxidation at high concentrations, but XL-8-NPs revealed a significantly higher activity (Figure 6D). It is likely that the endocytic method of XL-8-NPs internalization, utilizing a lot of membrane components, promoted lipid peroxidation in contrast with free XL-8. Interestingly, XL-8 and XL-8-NPs at 60 nM decreased MDA levels to the reference value, which may be explained by the induction of proliferation under the mild oxidative conditions and activation of lipid synthesis [60].

Thus, both XL-8 and XL-8-NPs stimulated ROS formation, leading to mitochondrial failure, DNA fragmentations, GSH depletion, and lipid peroxidation, resulting in initiation of apoptosis [61].

### 3.13. In Vivo Antitumor Activity

**Figure 7:**
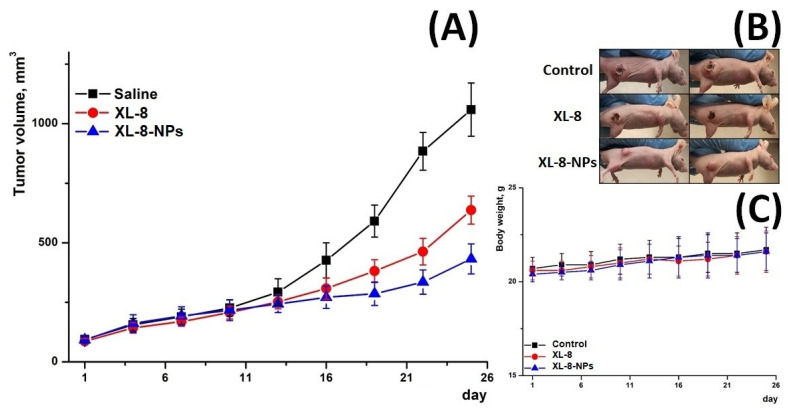
Tumor growth inhibition after treatment (**A**). Representative photo of treated tumor-bearing mice taken on day 25 (**B**). Body weight changes of tumor-bearing mice over the treatment period (**C**) (*n* = 3).

Furthermore, we evaluated in vivo antitumor activity of XL-8 and X-8-NPs (Figure 7) in an ectopic HeLa tumor-bearing mouse model. The therapeutic effect was assessed by measuring tumor volumes. All mice demonstrated rapid tumor growth after implantation. Upon irradiation, the XL-8 group displayed tumor growth inhibition. However, the most prominent suppressor effect was revealed in the group injected with XL-8-NPs.

These effects may be explained by the better intratumoral accumulation of NPs-based formulation due to enhanced permeability and retention effect and longer blood circulation time. In contrast, poor water solubility, aggregation, and rapid tissue distribution may significantly limit XL-8 bioavailability and activity. The consistent body weight in all groups evidenced a negligible systemic toxicity of XL-8 and XL-8-NPs.

## 4. Discussion

Modern medicine applies PDT as a noninvasive treatment of various cancers. The key components of PDT are PS and light source, which are under constant development in order to improve efficacy and safety of PDT [62]. The main problems of major PS are high lipophilicity and low tumor uptake efficiency, which complicate therapeutic PDT application: lipophilic PS may partially precipitate in organisms and adhere to the membranes of normal cells [63]. One of the most popular PSs applied today is Ce6. Researchers made plenty Ce6 modifications and formulations, including various nanoforms of Ce6, with increased specificity and photoactivity [64,65,66,67]. We improved the potential of therapeutic applicability of highly active Ce6 derivative, XL-8, via encapsulation in PLGA and assessed the properties and in vitro antitumor activity of the obtained formulation.

NPs allow us to overcome drawbacks of conventional drugs, such as poor solubility in water, low bioavailability, and photostability, etc. Spherical shape, negative surface charge, and small size of NPs promote intratumoral accumulation and endocytosis, while XL-8 encapsulation in polymer matrix improves photostability and regulates drug release [28]. The formulated XL-8-loaded NPs displayed sizes of about 200 nm and zeta potential of about –20 mV, which potentially promote tumor accumulation and avoid excretion by the kidney and absorption by macrophages.

One of the main advantages of NPs is a sustained release that prevents fast nonspecific drug distribution. The XL-8-NPs released about 50% of loaded XL-8 during the first 5 h, followed by a sustained release phase. Such a biphasic release profile provides a convenience in therapeutic applications that allows a reduced dose and administration frequency. Moreover, the PLGA matrix increased photostability of XL-8 under irradiation.

We analyzed the phototoxicity of XL-8 and XL-8-NPs against SK-OV-3, HeLa, A549, MCF-7, and 4T1 cell lines, where HeLa cells displayed prominent sensitivity. It was shown that XL-8 entrapment in PLGA matrix preserved PS activity. Colony formation assay revealed an enhanced XL-8-NPs antiproliferative activity over XL-8. According to CLSM data, XL-8-NPs prevalently distributed along the cytoplasm, especially in the perinuclear area, and colocalized partially with mitochondria, which agreed with the report by Li et al., which described the Ce6 specificity to mitochondria [68,69]. Interestingly, XL-8-NPs treated samples displayed a higher percentage of TUNEL-positive cells, which may be explained by the peculiarity of NPs internalization and perinuclear localization, promoting extensive DNA damage.

The main molecular mechanism of XL-8-induced PDT toxicity is based on oxidative stress [62]. Both XL-8 and XL-8-NPs after irradiation triggered ROS generation during the first 6 min, while XL-8-NPs stimulated higher levels of ROS generation; at 3 h after PDT treatment, XL-8 and XL-8-NPs demonstrated the maximal levels of ROS formation. Recent reports described that photoirradiation induces not only apoptosis but also ferroptosis, triggered by GSH depletion [70,71]. Indeed, short time XL-8 and XL-8-NPs treatment induced GSH depletion, suggesting ferroptosis as a possible cell death pathway induced by PDT [57,72,73]. MDA assay revealed an accumulation of lipid peroxidation products, which also indicated a ferroptosis [72]. Furthermore, we clarified the mechanism of XL-8 and XL-8-NPs toxicity, investigating the involvement in apoptosis. Both vehicles induced apoptosis at low concentrations with insignificant difference, according to annexin V-FITC/PI staining. However, high XL-8 concentration more effectively induced the formation of late apoptic/necrotic population, which may be explained with the differences in internalization kinetics and interaction with membrane compartments. Next, we analyzed mitochondrial depolarization, involved in apoptosis, resulting in cytochrome c release and subsequent cell death [56]. The XL-8 treated samples demonstrated a higher loss of MMP over XL-8-NPs treated sample, which may correlate with high lipophilicity and mitochondrial specificity of free PS.

These results revealed an activation of both apoptosis and ferroptosis pathways after XL-8 and XL-8-NPs-based PDT. Nevertheless, XL-8-NPs displayed more effective GSH depletion and lipid peroxidation, evidencing a stronger ferroptosis involvement, while free XL-8 provoked more prominent MMP loss and formation of late apoptic/necrotic populations, indicating that XL-8 induced apoptosis.

The evaluation of in vivo antitumor efficacy revealed a prominent XL-8-NPs activity over XL-8, which could be explained by a higher bioavailability of NPs-based vehicles.

Summarizing, we demonstrated a potential of the nanoformulation of a new Ce6 derivative, XL-8, for PDT, characterized by stable photoactivity and increased cell internalization. Utilization of the polymer matrix altered the intracellular distribution of XL-8 and promoted the damage of intracellular components. The level and character of oxidative damage induced by both vehicles involved apoptosis, as well as ferroptosis.

## 5. Conclusions

We successfully prepared XL-8-loaded PLGA NPs using the single emulsion-solvent evaporation method. XL-8-NPs revealed a spherical shape, small size, negative surface charge, and prolonged biphasic XL-8 release profile. Both XL-8 and XL-8-NPs demonstrated a high photoactivity via ROS (including mitochondrial superoxide) formation during the first 3 h after PDT treatment. XL-8 exhibited a stronger ability to depolarize mitochondria and induce apoptosis. In contrast, XL-8-NPs revealed a higher intracellular internalization and sustained XL-8 release, accompanied with more effective lipid peroxidation and GSH depletion, which indicated a greater ferroptosis involvement. Overall, these results demonstrated the potential of XL-8-loaded PLGA NPs application in PDT, promoting apoptosis and ferroptosis.

## Data Availability

Data is contained within the article.

## Supplementary Materials

The following are available online at https://www.mdpi.com/article/10.3390/antiox10121985/s1, Figure S1: DLS size distribution curve (A) and zeta potential (B) of XL-8-NPs, Figure S2: Graphical representation of the quantification of XL-8-NPs uptake by HeLa cells obtained from laser confocal scanning microscope, Table S1: Phototoxicity of XL-8 and XL-8-NPs on cell lines as determined by MTT assay. Results are presented as mean ± S.D. of triplicate of three independent experiments.
