# Supplementary material for: Oxidative Damage Induced by Phototoxic Pheophorbide a 17-Diethylene Glycol Ester Encapsulated in PLGA Nanoparticles
Source: Antioxidants (Basel). 2021 Dec 13;10(12):1985. doi: 10.3390/antiox10121985 (PMC8750000; doi:10.3390/antiox10121985)
Supplement: Supplementary file 1 [file antioxidants-10-01985-s001.zip › antioxidants-1451843-supplementary.pdf]

SUPPLEMENTARY MATERIALS

Oxidative Damage Induced by Phototoxic Pheophorbide a 17-Diethylene Glycol Ester Encapsulated in PLGA Nanoparticles

Mariia R. Mollaeva [1,2,*], Elena Nikolskaya [1,2], Maria Sokol [1,2], Veronika Beganovskaya [2,3], Margarita Chirkina [1,2], Murad D. Mollaev [4], Sergey Obydennyi [4,5], Dmitry Belykh [6], Olga Startseva [7], Nikita Yabbarov [1,2,*]

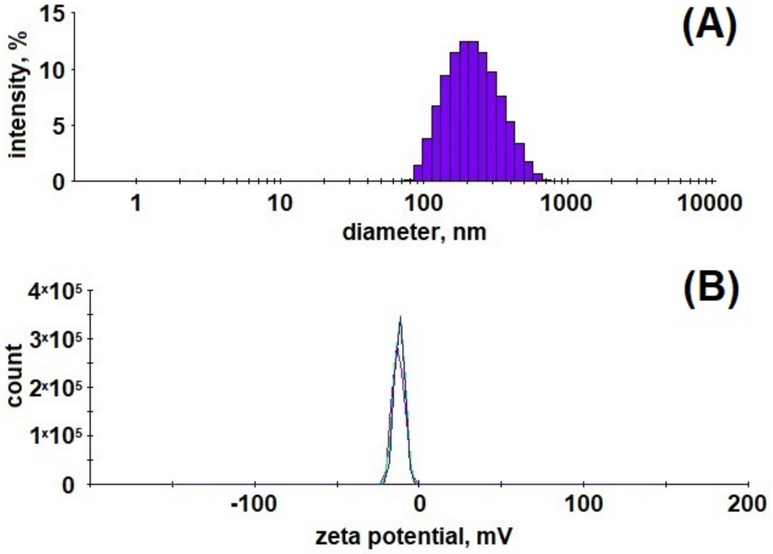

Figure S1. DLS size distribution (A) and zeta potential (B) of XL-8-NPs.

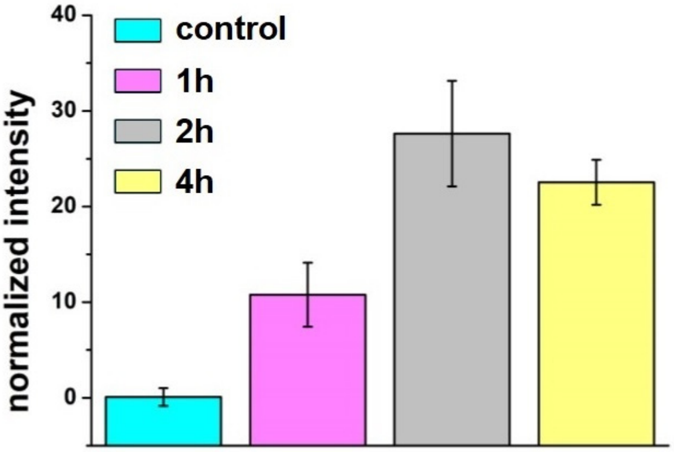

Figure S2. Graphical representation of the quantification of XL-8-NPs uptake by HeLa cells obtained from laser confocal scanning microscope.

Table S1. Phototoxicity of XL-8 and XL-8-NPs on cell lines as determined by MTT assay. Results are presented as mean ± S.D. of triplicate of three independent experiments.

Drug	**SK-OV-3**	**A549**	**HeLa**	**MCF7**	**4T1**
	IC50, nM				
**XL-8**	82.7±1.4	104.1±2.8	146.5±5.7	279.0±6.5	259.3±7.8
**XL-8-NPs**	106.6±4.6	150.4±6.3	47.6±3.1	217.5±5.4	252.2±8.3
